# High Humidity Response of Sol–Gel-Synthesized BiFeO_3_ Ferroelectric Film

**DOI:** 10.3390/ma15082932

**Published:** 2022-04-17

**Authors:** Yaming Zhang, Bingbing Li, Yanmin Jia

**Affiliations:** 1 School of Science, Xi’an University of Posts and Telecommunications, Xi’an 710048, China; zymon@163.com; 2 School of Communication and Information Engineering, Xi’an University of Posts and Telecommunications, Xi’an 710048, China; lbbing33@163.com

**Keywords:** ferroelectrics, BiFeO_3_, humidity response, sol–gel preparation

## Abstract

In this work, a BiFeO_3_ film is prepared via a facile sol–gel method, and the effects of the relative humidity (RH) on the BiFeO_3_ film in terms of capacitance, impedance and current–voltage (*I*–*V*) are explored. The capacitance of the BiFeO_3_ film increased from 25 to 1410 pF with the increase of RH from 30% to 90%. In particular, the impedance varied by more than two orders of magnitude as RH varied between 30% and 90% at 10 Hz, indicating a good hysteresis and response time. The mechanism underlying humidity sensitivity was analyzed by complex impedance spectroscopy. The adsorption of water molecules played key roles at low and high humidity, extending the potential application of ferroelectric BiFeO_3_ films in humidity-sensitive devices.

## 1. Introduction

Humid environments are essential in many fields, such as weather forecasting, agricultural production and personnel health [1,2]. In addition, trace amounts of water molecules can have a significant impact on industry and manufacturing [3,4]. Therefore, it is necessary to explore highly efficient and accurate humidity sensors. Humidity is a physical quantity that indicates the molecular content of water in the air, and is mainly measured by relative humidity. Over the past decade, many techniques for measuring humidity have been reported, including wet and dry bulb hygrometers, piezoelectric quartz films, resistive sensors, and sensors based on current, impedance and surface acoustic waves [2,5]. Among them, impedance-based humidity-sensing technology is the most convenient and commonly used [2]. Impedance humidity sensors work on the principle that changes in humidity can be reflected by changes in the impedance of a hygroscopic medium [6]. Impedance-type humidity sensors have been extensively reported in recent years due to their low cost, fast response speed and small size [6,7]. Impedance measurements indicate that suitable humidity-sensitive materials mainly include polymers, carbon materials and ceramic materials [8,9]. However, polymer films are not suitable for application at high temperatures. Ceramic films with good stability at high temperatures are considered to be the preferred materials for impedance-based humidity sensors due to their unique structure of grain boundaries, grains and pores [10]. 

For the convenience of microelectronics integration, film materials are often prepared for humidity sensors. Some ferroelectric perovskite (ABO_3_, where A is a rare earth, alkali or alkaline earth metal and B is a transition metal) humidity-sensing film materials, including BaTiO_3_, K_0.5_Bi_0.5_TiO_3_, K_0.5_Na_0.5_NbO_3_ and LaFeO_3_, can behave with remarkable humidity-sensing properties [11,12,13]. BiFeO_3_ is a well-known lead-free ferroelectric material that has been regarded as a promising spintronic and information-storage receptor material in recent years due to its large remanent polarization and high Curie point [14,15,16]. BiFeO_3_ is a distorted perovskite ferroelectric material with a non-stoichiometric ratio, which makes it behave with *p*-type semiconductor behavior and makes it a promising material for high-performance humidity-sensing applications [17,18]. So far, there are few reports exploring the humidity-sensing behavior of BiFeO_3_ films. In humidity sensors, morphology and cation distribution can be controlled by the synthesis method, which affects the surface reaction. The sol–gel technique is a simple, low-cost and promising method for the preparation of BiFeO_3_ films [19].

In this work, the capacitance of BiFeO_3_ film synthesized via the sol–gel method was found to increase from 25 to 1410 pF when RH increased from 30% to 90%. In particular, the impedance varied by more than two orders of magnitude when RH varied between 30% and 90% at 10 Hz, which extends the potential application of ferroelectric BiFeO_3_ films to humidity-sensitive devices.

## 2. Materials and Methods

The sol–gel method was used to successfully prepare a BiFeO_3_ film. Powders of bismuth nitrate (Bi(NO_3_)_3_·5H_2_O) and ferric nitrate (Fe(NO_3_)_3_·9H_2_O) were dissolved in C_3_H_8_O_2_ solution with a molar ratio of 1:1 and agitated at room temperature for 30 min. Afterwards, enough CH_3_COOH was added to the solution for dehydration. During continuous stirring, 5 mL of aminoethanol was added to BiFeO_3_ solution in order to control the viscosity. Finally, a 0.3 mol/L red-brown mixed solution with a volume of 30 mL was obtained. The mixture was stirred on a magnetic stirrer for 2 h and left at room temperature for 12 h. The obtained reddish-brown BiFeO_3_ solution was spin-coated on a Pt/Si(111) substrate and dried for 3 min at 180 °C. Then, films were calcined for 20 min at 490 °C. Finally, conductive silver glue was used to stick electrodes on the surface of the BiFeO_3_ film for the electrical measurement.

The simple structure was determined via XRD (D/Max2550VB+/PC, Japan). The microstructure was characterized via SEM (Nova NanoSEM 450, Lincoln, NE, USA). A ferroelectric analyzer was used to explore the ferroelectric hysteresis loop (Precision Multiferroic, Radiant Technology, Albuquerque, NM, USA). The capacitance and impedance were measured using a precision impedance analyzer (Novocontrol GmbH, Montabaur, Germany). The current–voltage relationship was measured using a current–voltage meter (Agilent B2902A, Santa Clara, CA, USA). A humidifier in an enclosed space was employed to generate an environment with 30% to 90% relative humidity. The RH was measured using a hygrometer.

## 3. Results and Discussion

### 3.1. Structure and Morphology of Material

**Figure 1:**
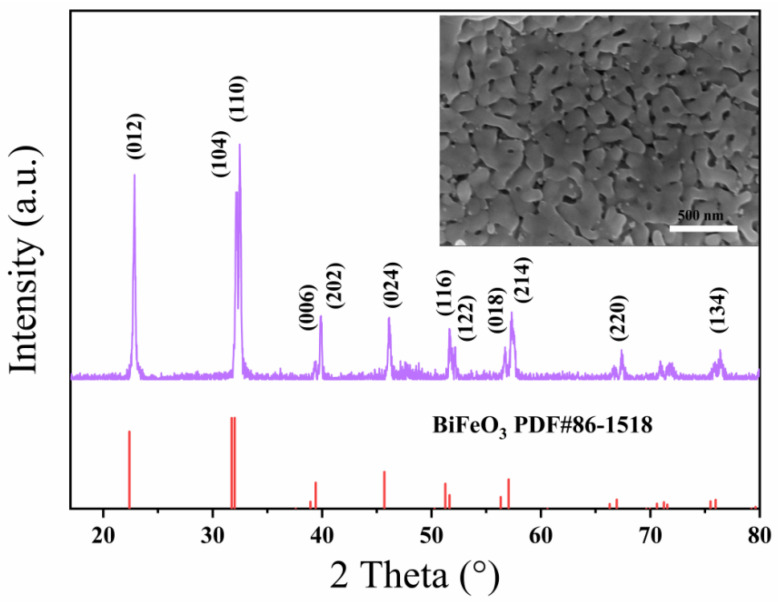
XRD pattern of BiFeO_3_ film. Inset: SEM image of BiFeO_3_ film.

Figure 1 shows the X-ray diffractometer (XRD) patterns of the BiFeO_3_ film. The diffraction peaks of the pure BiFeO_3_ sample are consistent with the standard chart of BiFeO_3_ with rhombohedral *R*3c structure (JCPDS PDF # 86-1518), as shown in Figure 1. There is no impurity peak, which proves that the sample is a pure-phase perovskite structure BiFeO_3_. The scanning electron microscope image of the BiFeO_3_ film is shown in the inset of Figure 1. The image reveals that the as-synthesized BiFeO_3_ film had a porous structure, indicating its excellent ability to adsorb water molecules, which is essential for humidity sensing.

**Figure 2:**
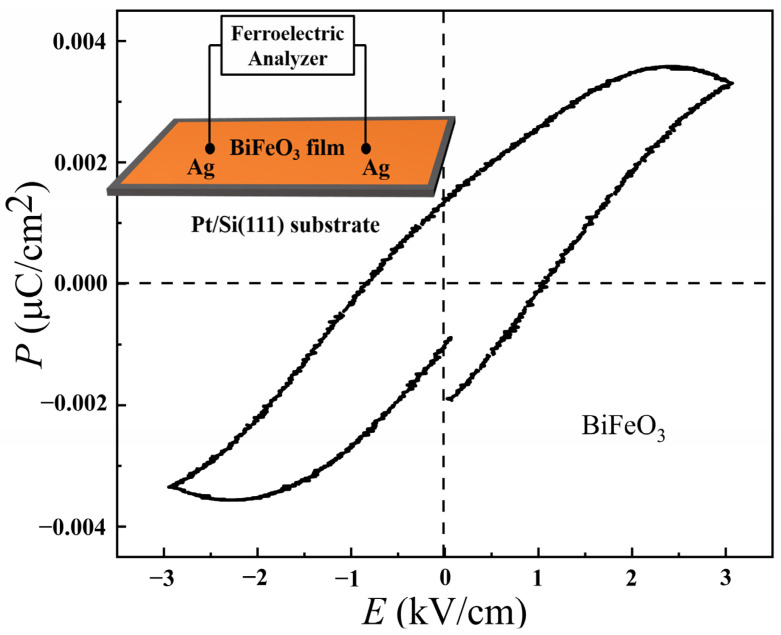
Ferroelectric hysteresis loop. Inset: schematic diagram of the test circuit.

The ferroelectric hysteresis loop of the BiFeO_3_ film is shown in Figure 2. A schematic diagram of the ferroelectric test circuit is shown in the inset of Figure 2. This test circuit was composed of two silver electrode points coated on the surface of the material to connect the wires. In Figure 2, the unsaturated ferroelectric hysteresis loop was obtained due to the serious leakage current [19]. The composition of the sample indicates that the BiFeO_3_ film had a serious electrical leakage problem due to the multiple valence states of Fe [19].

### 3.2. Humidity-Sensing Properties

**Figure 3:**
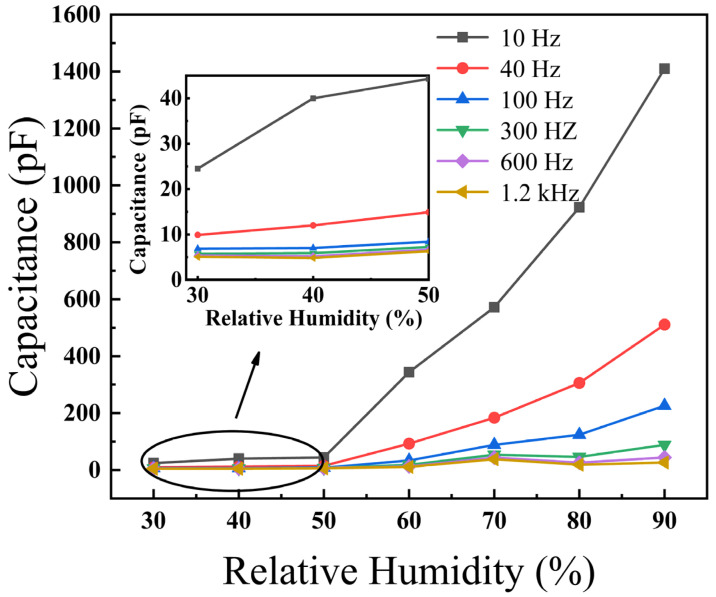
RH dependence on the capacitance of BiFeO_3_ film. Inset: enlarged capacitance vs. %RH plot (30–50% RH range).

The dependence of the capacitance of the BiFeO_3_ film on the RH was measured at the frequencies of 10, 40, 100, 300, 600 and 1200 Hz, as shown in Figure 3. The inset is a partial enlarged view of capacitance change with RH (RH 30−50%) at different frequencies. At low frequency (i.e., 10 Hz, 40 Hz, 100 Hz), the capacitance increased significantly with increasing RH. In particular, the capacitance of the BiFeO_3_ film increased from 25 to 1410 pF as the RH increased from 30% to 90% at 10 Hz. This was due to the increase of physisorbed water molecules on the BiFeO_3_ film surface with the increase of RH, which made more water molecules polarized. At high frequency (i.e., 300 Hz, 600 Hz, 1200 Hz), the capacitance remained almost constant with increasing RH, implying that frequency is a crucial factor in the humidity response. At high frequency, the dipoles of the water molecules slow their reorientation. The dipole rotation of water molecules no longer resonates with the external field at high frequencies, which means that the polarizability of the water molecules lags behind the frequency of the change of the external electric field. Therefore, the capacitance of the BiFeO_3_ film had a high humidity response at frequencies range of 10–100 Hz, while RH is independent of the capacitance at frequencies in the range of 100 Hz to 1.2 kHz. The effect of RH on capacitance can be expressed by Equation (1) [20]

(1) $$C=(\varepsilon_{\gamma }-i\times \frac{\gamma }{\omega \times \varepsilon_{0}})\times C_{0}$$

where *ε_γ_* and *γ* are the permittivity and the electrical conductivity of the BiFeO_3_ film, respectively. *C*_0_ and *ε*_0_ denote the capacitance of an ideal capacitor and the vacuum permittivity, respectively. *C* and *ω* are the capacitance and the frequency, respectively. Equation (1) indicates that the capacitance of the BiFeO_3_ film is inversely related to *ω* and is proportional to the material’s *γ*. Both *γ* and *C* increase as RH increases [20]. 

**Figure 4:**
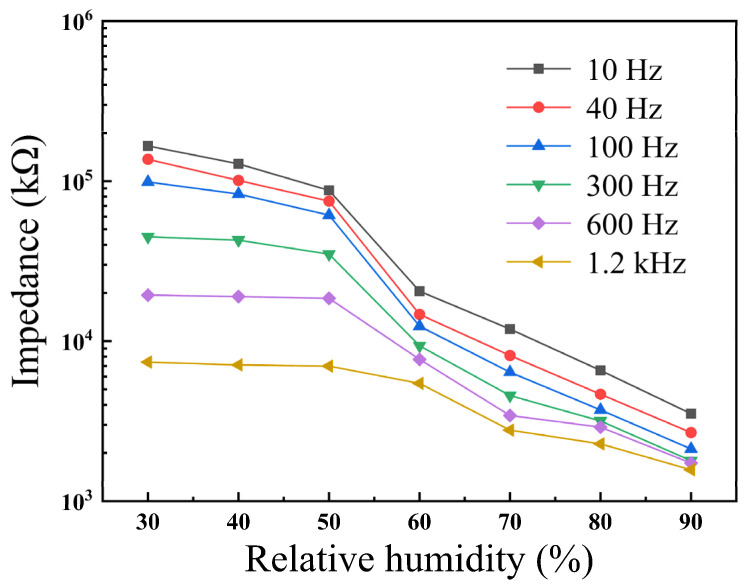
RH dependence on the impedance of BiFeO_3_ film.

In order to determine the optimal working frequency, the dependence of impedance on RH was measured using BiFeO_3_ film at 30–90% RH and frequencies of 10, 40, 100, 300, 600 and 1200 Hz, as shown in Figure 4. Since it is difficult to lead the adsorbed water molecules to modify the associated polarization at high frequencies, there was a weak response to humidity at these frequencies. Therefore, it is important to determine the optimal frequency for RH measurements [21]. Figure 4 shows that the impedance of the BiFeO_3_ film decreased from 1.7 × 10^5^ to 1570 kΩ when RH increased from 30% to 90%. The impedance decreased significantly at 10 Hz, indicating that the optimum working frequency is 10 Hz. Over the entire frequency range, the impedance decreased with the increase of RH. At the same frequency, the impedance change was not obvious at low RH, while the impedance drop was more significant at high RH. This is because the main conduction mechanism for humidity sensing is caused by proton hopping between the sensitive layer of the film and water molecules. At low RH, a small amount of water molecules are chemisorbed on the cations (Bi^3+^ and Fe^3+^) on the film surface [22]. Due to the lack of a complete adsorption layer, the low polarizability of water molecules eventually leads to high impedance. At high RH, multiple layers of physical adsorption are formed on the basis of the chemical adsorption layer, resulting in the movement of more protons in the water layer [22]. This results in a significant increase in the conductivity of the humidity sensor and a decrease in impedance. 

**Figure 5:**
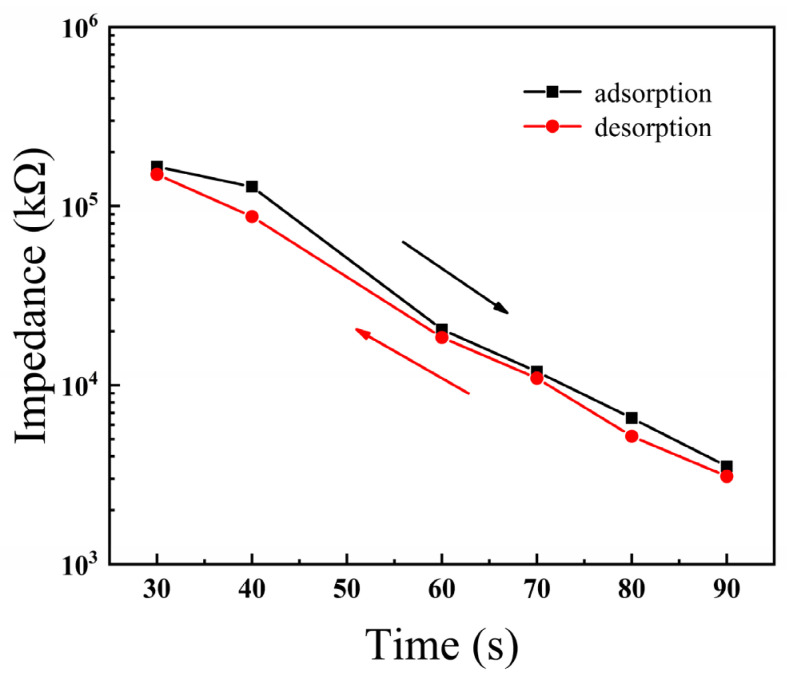
Humidity hysteresis characteristics of BiFeO_3_ film measured at 10 Hz.

Humidity hysteresis of the BiFeO_3_ film usually occurred during the desorption of samples. The humidity hysteresis is a critical characteristic for the application of humidity sensing, and is defined as the maximum difference between adsorption and desorption of the humidity sensor. The humidity hysteresis (*γH*) is expressed in Equation (2) as [21]:

(2) $$\gamma H=\pm \frac{\Delta RH_{MAX}}{2F_{FS}}$$

where *RH_MAX_* is the maximum difference in the output of adsorption and desorption processes. *F_FS_* is the impedance change over the entire humidity range. The humidity hysteresis characteristics of the BiFeO_3_ humidity sensor at 10 Hz are shown in Figure 5. It can be seen from the figure that the BiFeO_3_ showed a narrow hysteresis loop. The BiFeO_3_ film had a small hysteresis during the entire humidity test with a maximum hysteresis of approximately 16%, mainly caused by residual moisture in the BiFeO_3_ film layer. With the decrease of RH, the number of water molecules between the layers of the BiFeO_3_ film gradually decreased, resulting in the gradual disappearance of the hysteresis phenomenon [23,24].

**Figure 6:**
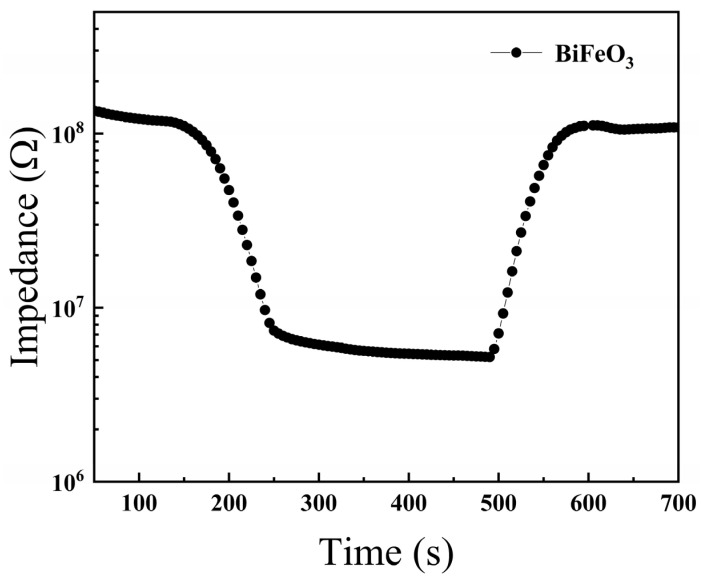
Humidity response and recovery curve of BiFeO_3_ film measured at 10 Hz.

Based on the conversion circuit of a humidity sensor, RH changes in the environment can be converted into an electrical signal that is easy to control and identify. The ideal humidity sensor needs to meet the following characteristics: fast response speed, strong recovery ability and small humidity hysteresis error. The response and recovery times are the times required for the BiFeO_3_ film to reach 90% of the total impedance change during adsorption and desorption, respectively. Figure 6 shows that the humidity response and recovery times of the BiFeO_3_ film in the maximum humidity range (30–90% RH) were 60 s and 70 s at 10 Hz, respectively. The recovery time of the BiFeO_3_ film was higher than the response time due to the higher bonding energy between the adsorbed water molecules and the surface of the sensor material [25]. This result indicates that the BiFeO_3_ film could rapidly adsorb and desorb water molecules, indicating its potential value for practical applications. 

### 3.3. Humidity-Sensing Mechanism

**Figure 7:**
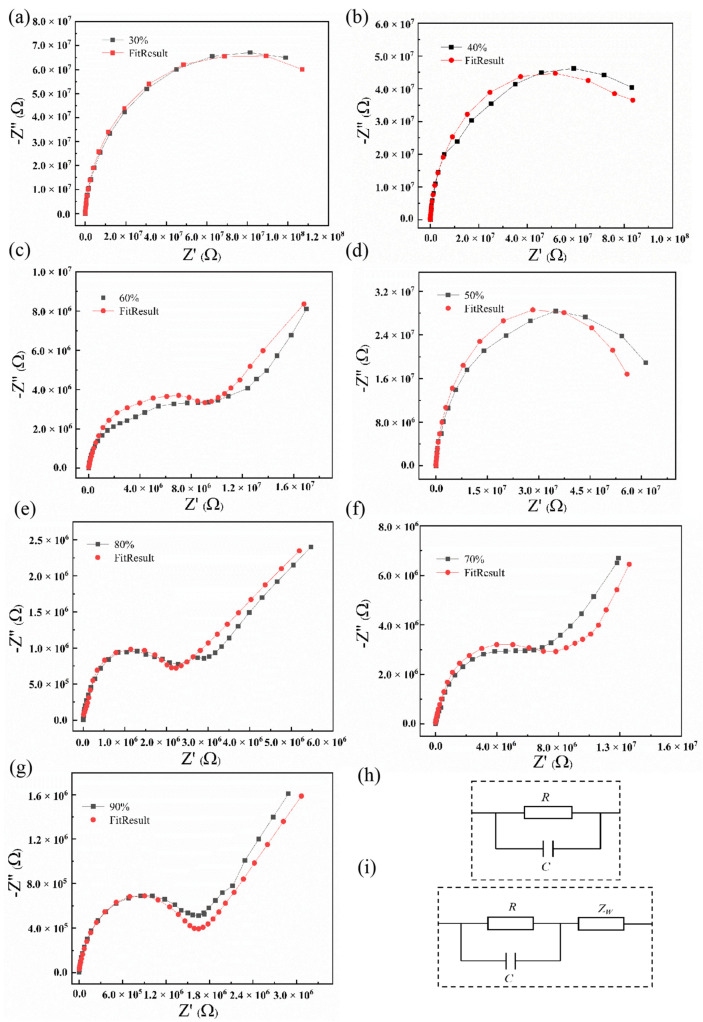
The complex impedance properties of BiFeO_3_ film under different humidities. (**a**) 30% RH; (**b**) 40% RH; (**c**) 50% RH; (**d**) 60% RH; (**e**) 70% RH; (**f**) 80% RH; (**g**) 90% RH. (**h**) The equivalent circuit fit by Zview in the 30−50% RH range. (**i**) The equivalent circuit fit by Zview in the 60−90% RH range.

The complex impedance curve is an effective method to study the properties of humidity sensing [26]. In AC complex impedance analysis, an AC sinusoidal test signal is applied to a thin-film device, and the frequency of the test signal is changed within a certain range. Figure 7 shows the complex impedance spectrum of the BiFeO_3_ film in the range of 30–90% RH and in the scanning frequency range of 10–1000 kHz. The complex impedance spectrum of the BiFeO_3_ film presented a circular arc shape when the RH was lower than 50%, as shown in Figure 7a–c. The complex impedance spectrum gradually changed from a circular arc to a semicircular shape with increasing humidity. Compared to the complex impedance spectra of standard circuit components, it can be concluded that the equivalent circuit diagram for BiFeO_3_ films in the low-humidity range is composed of parallel connections of resistors and capacitors, as shown in Figure 7h. Oxygen ions and metal ions are exposed on the surface of the BiFeO_3_ film, and the H_2_O molecules on the surface dissociate into H^+^ and OH^−^. Then, OH^−^ and H^+^ are chemically combined with metal ions and oxygen ions, respectively, to form hydroxyl groups that constitute the first layer of physical adsorption [23]. The charge transfer is carried out according to the Grotthuss chain reaction of 2 H_2_O → H_3_O^+^ + OH^−^, which has a weak influence on the capacitance of the BiFeO_3_ film. H_3_O^+^ spontaneously transfers H^+^ to the second water molecule according to H_3_O^+^ → H_2_O + H^+^ [27,28], and the main mechanism underlying the humidity response is based on proton transport [29].

When RH increased to 70%, the complex impedance spectrum of the BiFeO_3_ film showed a straight line with a slope of approximately 1 at frequencies from 10 to 100 Hz, as shown in Figure 7d,e. On top of the first layer of physical adsorption, more adsorption layers are formed through hydrogen bonding to generate a liquid water layer, and the physical adsorption changes from single-layer to multi-layer [30]. When RH increased to 90%, the proportion of the straight-line part of the complex impedance spectrum increased, while the semicircle part was compressed. The appearance of a straight line in the low-frequency region of the complex impedance spectrum indicates that the BiFeO_3_ film has a significant Warburg impedance due to ion diffusion, as shown in Figure 7f,g. The corresponding equivalent circuit includes resistance, capacitance and Warburg impedance, as shown in Figure 7i. With the continuous increase in the number of adsorbed water molecules, the adsorption on the sample surface evolves into multi-molecular layer adsorption. The surface of the BiFeO_3_ films is covered by water, resulting in a rapid increase in the amount of H^+^, which further increases the conductivity [31,32].

**Figure 8:**
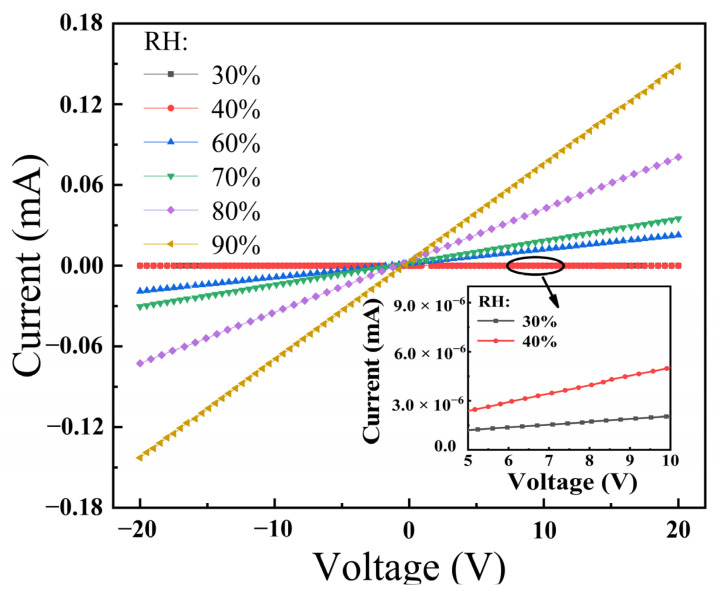
Dependence of current on voltage for the BiFeO_3_ film at various RHs. Inset: the enlarged *I*–*V* vs. %RH plot (30–40% RH range).

The *I*–*V* characteristics of the BiFeO_3_ film at different RH levels are presented in Figure 8. The inset is a partial enlarged view of the change in *I*–*V* with RH. At different RHs, BiFeO_3_ film exhibited linear *I*–*V* characteristics, which indicates an ohmic contact between the BiFeO_3_ film surface and electrodes. Since the resistance was constant over the range of supply voltage, the sensitivity was the same regardless of the operating bias, which allows operation at low power in practical application [33]. As RH increased, the conductivity of the BiFeO_3_ film increased, resulting in a decrease in current. The excellent humidity response makes BiFeO_3_ films a potential candidate for practical humidity-sensing applications.

## 4. Conclusions

The BiFeO_3_ film prepared in this study via a simple sol–gel method exhibited significant humidity sensitivity with capacitance and impedance changes of nearly 2–3 orders of magnitude as RH increased from 30% to 90%. In the whole humidity range, the experimental results of humidity hysteresis and humidity response recovery indicate that BiFeO_3_ film is an excellent material for application in humidity sensors.

## Data Availability

The data presented in this study are available on request from the corresponding author.

## References

[1] Qi R., Lin X., Dai J., Zhao H., Liu S., Fei T., Zhang T. (2018). Humidity sensors based on MCM-41/polypyrrole hybrid film via in-situ polymerization. Sens. Actuators B Chem..

[2] Zhang Y., Duan Z., Zou H., Ma M. (2018). Drawn a facile sensor: A fast response humidity sensor based on pencil-trace. Sens. Actuators B Chem..

[3] Si R.J., Li T.Y., Sun J., Wang J., Wang S.T., Zhu G.B., Wang C.C. (2019). Humidity sensing behavior and its influence on the dielectric properties of (In + Nb) co-doped TiO_2_ ceramics. J. Mater. Sci..

[4] Wang J., Guo Y.M., Wang S.T., Tong L., Sun J., Zhu G.B., Wang C.C. (2019). The effect of humidity on the dielectric properties of (In+Nb) co-doped SnO_2_ ceramics. J. Eur. Ceram. Soc..

[5] Duan Z., Zhao Q., Wang S., Yuan Z., Zhang Y., Li X., Wu Y., Jiang Y., Tai H. (2020). Novel application of attapulgite on high performance and low-cost humidity sensors. Sens. Actuators B Chem..

[6] Wu Z., Yang J., Sun X., Wu Y., Wang L., Meng G., Kuang D., Guo X.Z., Qu W., Du B. (2021). An excellent impedance-type humidity sensor based on halide perovskite CsPbBr_3_ nanoparticles for human respiration monitoring. Sens. Actuators B Chem..

[7] Weng Z., Qin J., Umar A.A., Wang J., Zhang X., Wang H., Cui X., Li X., Zheng L., Zhan Y. (2019). Lead-free Cs_2_BiAgBr_6_ double perovskite-based humidity sensor with superfast recovery time. Adv. Funct. Mater..

[8] Cho M.-Y., Kim S., Kim I.-S., Kim E.-S., Wang Z.-J., Kim N.-Y., Kim S.-W., Oh J.-M. (2020). Perovskite-induced ultrasensitive and highly stable humidity sensor systems prepared by aerosol deposition at room temperature. Adv. Funct. Mater..

[9] Dai J., Zhang T., Zhao H., Fei T. (2017). Preparation of organic-inorganic hybrid polymers and their humidity sensing properties. Sens. Actuators B Chem..

[10] Farahani H., Wagiran R., Urban G.A. (2020). Investigation of room temperature protonic conduction of perovskite humidity sensors. IEEE Sens. J..

[11] Kumar A., Wang C., Meng F.-Y., Liang J.-G., Xie B.-F., Zhou Z.-L., Zhao Z., Kim N.-Y. (2021). Aerosol deposited BaTiO_3_ film based interdigital capacitor and squared spiral capacitor for humidity sensing application. Ceram. Int..

[12] Zhao J., Liu Y., Li X., Lu G., You L., Liang X., Liu F., Zhang T., Du Y. (2013). Highly sensitive humidity sensor based on high surface area mesoporous LaFeO_3_ prepared by a nanocasting route. Sens. Actuators B Chem..

[13] Wang N., Luo X., Han L., Zhang Z., Zhang R., Olin H., Yang Y. (2020). Structure, performance, and application of BiFeO_3_ nanomaterials. Nano-Micro Lett..

[14] Yang S.Y., Zhang F., Xie X., Sun H., Zhang L., Fan S. (2018). Enhanced leakage and ferroelectric properties of Zn-doped BiFeO_3_ thin films grown by sol-gel method. J. Alloy Compd..

[15] Chen M., Jia Y., Li H., Wu Z., Huang T., Zhang H. (2021). Enhanced pyrocatalysis of the pyroelectric BiFeO_3_/g-C_3_N_4_ heterostructure for dye decomposition driven by cold-hot temperature alternation. J. Adv. Ceram.

[16] Wu J., Mao W., Wu Z., Xu X., You H., Xue A., Jia Y.A.X. (2016). Strong pyro-catalysis of pyroelectric BiFeO_3_ nanoparticles under a room-temperature cold-hot alternation. Nanoscale.

[17] You H., Jia Y., Wu Z., Xu X., Qian W., Xia Y., Ismail M. (2017). Strong piezo-electrochemical effect of multiferroic BiFeO_3_ square micro-sheets for mechanocatalysis. Electrochem. Commun..

[18] Preethi A.J., Ragam M. (2021). Effect of doping in multiferroic BFO: A review. J. Adv. Dielect..

[19] Xu X., Xiao L., Haugen N.O., Wu Z., Jia Y., Zhong W., Zou J. (2018). High humidity response property of sol-gel synthesized ZnFe_2_O_4_ films. Mater. Lett..

[20] Liang J.-G., Kim E.-S., Wang C., Cho M.-Y., Oh J.-M., Kim N.-Y. (2018). Thickness effects of aerosol deposited hygroscopic films on ultra-sensitive humidity sensors. Sens. Actuators B Chem..

[21] Gong M., Li Y., Guo Y., Lv X., Dou X. (2018). 2D TiO_2_ nanosheets for ultrasensitive humidity sensing application benefited by abundant surface oxygen vacancy defects. Sens. Actuators B Chem..

[22] Rachida D., Nouara L., M’hand O., Malika S., Yannick G., Ahcène C., Bertrand B. (2020). Improvement of humidity sensing performance of BiFeO_3_ nanoparticles-based sensor by the addition of carbon fibers. Sens. Actuator A Phys..

[23] Mahapatra P.L., Das S., Mondal P.P., Das T., Saha D., Pal M. (2021). Microporous copper chromite thick film based novel and ultrasensitive capacitive humidity sensor. J. Alloy Compd..

[24] Duan Z., Zhao Q., Wang S., Huang Q., Yuan Z., Zhang Y., Jiang Y., Tai H. (2020). Halloysite nanotubes: Natural, environmental-friendly and low-cost nanomaterials for high-performance humidity sensor. Sens. Actuators B Chem..

[25] Liang J.-G., Wang C., Yao Z., Liu M.-Q., Kim H.-K., Oh J.-M., Kim N.-Y. (2018). Preparation of ultrasensitive humidity-sensing films by aerosol deposition. ACS Appl. Mater. Interfaces.

[26] Zia T.H., Ali Ahsh A.H. (2021). Understanding the adsorption of 1 NLB antibody on polyaniline nanotubes as a function of zeta potential and surface charge density for detection of hepatitis C core antigen: A label-free impedimetric immunosensor. Colloids Surf. A Physicochem. Eng. Asp..

[27] Li T.Y., Si R.J., Sun J., Wang S.T., Wang J., Ahmed R., Zhu G.B., Wang C.C. (2019). Giant and controllable humidity sensitivity achieved in (Na+ Nb) co-doped rutile TiO_2_. Sens. Actuators B Chem..

[28] Si R., Xie X., Li T., Zheng J., Cheng C., Huang S., Wang C. (2020). TiO_2_/(K, Na)NbO_3_ nanocomposite for boosting humidity-sensing performances. ACS Sens..

[29] Mallick S., Ahmad Z., Qadir K.W., Rehman A., Shakoor R.A., Touati F., Al-Muhtaseb S.A. (2020). Effect of BaTiO_3_ on the sensing properties of PVDF composite-based capacitive humidity sensors. Ceram. Int..

[30] Nikolic M.V., Krstic J.B., Labus N.J., Lukovic M.D., Dojcinovic M.P., Radovanovic M., Tadic N.B. (2020). Structural, morphological and textural properties of iron manganite (FeMnO_3_) thick films applied for humidity sensing. Mater. Sci. Eng. B.

[31] Ji G.-J., Zhang L.-X., Zhu M.-Y., Li S.-M., Yin J., Zhao L.-X., Fahlman B.D., Bie L.-J. (2018). Molten-salt synthesis of Ba_5−*x*_Sr*_x_*Nb_4_O_15_ solid solutions and their enhanced humidity sensing properties. Ceram. Int..

[32] Ma H., Fang H., Wu W., Zheng C., Wu L., Wang H. (2020). A highly transparent humidity sensor with fast response speed based on α-MoO_3_ thin films. RSC Adv..

[33] Han J.-W., Kim B., Li J., Meyyappan M. (2012). Carbon nanotube based humidity sensor on cellulose paper. J. Phys. Chem. C..

